# Incidence of Fractures Before and After Dialysis Initiation

**DOI:** 10.1002/jbmr.4141

**Published:** 2020-09-16

**Authors:** Ken Iseri, Juan Jesús Carrero, Marie Evans, Li Felländer‐Tsai, Hans E Berg, Björn Runesson, Peter Stenvinkel, Bengt Lindholm, Abdul Rashid Qureshi

**Affiliations:** ^1^ Divisions of Renal Medicine and Baxter Novum, Department of Clinical Science, Intervention and Technology Karolinska Institutet Stockholm Sweden; ^2^ Division of Nephrology, Department of Medicine Showa University School of Medicine Tokyo Japan; ^3^ Department of Medical Epidemiology and Biostatistics Karolinska Institutet Stockholm Sweden; ^4^ Division of Orthopaedics and Biotechnology, Department of Clinical Science, Intervention and Technology Karolinska Institutet Stockholm Sweden

**Keywords:** DIALYSIS, END‐STAGE KIDNEY DISEASE, FRACTURE, RENAL OSTEODYSTROPHY

## Abstract

Fractures are common in dialysis patients, but little is known about the trajectory of incidence rates of different types of fractures before and after dialysis initiation. To address this, we investigated the incidence of major fractures before and after dialysis initiation. We performed a retrospective statistical analysis using the Swedish Renal Registry of 9041 incident dialysis patients (median age 67 years, 67% men) starting dialysis 2005 through 2015 to identify major fractures (hip, spine, humerus, and forearm) occurring during the dialysis transition period from 1 year before until 1 year after dialysis initiation. Using flexible parametric hazard models and the Fine‐Gray model, we estimated adjusted fracture incidence rates and predictors of major fractures. We identified 361 cases with primary diagnosis of major fracture, of which 196 (54%) were hip fractures. The crude incidence rate of major fractures before dialysis initiation was 17 per 1000 patient‐years (*n* = 157) and after dialysis initiation it was 24 per 1000 patient‐years (*n* = 204). The adjusted incidence rate of major fractures began to increase 6 months before dialysis initiation, and then stabilized at a higher rate after 1 year. The adjusted incidence rate of hip fractures started to increase sharply 3 months before dialysis initiation, peaked at initiation, and declined thereafter. In contrast, the adjusted incidence rate of non‐hip fractures was stable during the transition period and gradually increased over time. Higher age, female sex, and history of previous major fractures were associated with increased fracture incidence both before and after dialysis initiation. We conclude that the incidence of major fractures, especially hip fractures, start to rise 6 months before initiation of dialysis therapy, indicating that heightened surveillance with implementation of preventive measures to avoid fractures is warranted during the transition period to dialysis. © 2020 The Authors. *Journal of Bone and Mineral Research* published by Wiley Periodicals LLC on behalf of American Society for Bone and Mineral Research (ASBMR).

## Introduction

Chronic kidney disease (CKD) is common, associates with increased morbidity and mortality, and is now recognized as a major global health problem. Renal osteodystrophy caused by deterioration of kidney function is a frequent complication that may contribute to an increased fracture risk. Among non‐dialysis–dependent CKD patients, fracture risk, especially for hip fractures, increases as the estimated glomerular filtration rate (eGFR) declines.^(^ ^1^ ^)^ Dialysis patients have a markedly elevated risk of fractures compared with non‐dialyzed CKD patients, kidney transplanted patients, and the general population.^(^ ^2^, ^3^, ^4^ ^)^ The increased fracture risk may conceivably be explained, at least partially, by deterioration of physical functions, by frailty and falls linked to hemodynamic changes, and by poor nutritional status and metabolic alterations due to CKD–mineral bone disorder (CKD‐MBD).^(^ ^2^, ^5^, ^6^ ^)^

When kidney function is severely compromised, pronounced phenotypic and pathophysiologic changes occur during the transition period from non‐dialysis phase to dialysis phase^(^ ^7^ ^)^ including deterioration of physical and functional performance.^(^ ^8^, ^9^ ^)^ Increased morbidity following fractures, especially hip fractures, and subsequent high mortality are well described in the general population, non‐dialyzed CKD patients, and dialysis patients.^(^ ^1^, ^10^, ^11^, ^12^, ^13^, ^14^, ^15^ ^)^ Although a higher fracture risk in dialysis patients compared to CKD patients is well documented,^(^ ^2^, ^3^, ^16^ ^)^ there has been no investigation showing the impact of dialysis initiation and the period preceding dialysis initiation on fracture event rate. Because deterioration of physical functions occurs already before initiation of dialysis, we postulate that fracture events are affected not only by initiation of dialysis per se but also by changes during the pre‐dialysis period. A better understanding of the temporal changes of incident fractures and their predictors during the transition period is warranted because this could lead to interventions aiming at preventing fractures and improving clinical outcomes including quality of life.

The aim of the present study was to investigate the sequential pattern of fracture incidence rates in conjunction with the transition period of dialysis initiation, beginning 1 year before dialysis initiation and ending 1 year after. We also explored predictors of fracture events during the non‐dialysis phase and the period following dialysis initiation.

## Patients and Methods

### Patients and data sources

Using data from the Swedish Renal Registry (SRR), a nationwide registry of patients on renal replacement therapy (RRT) in Sweden^(^ ^17^ ^)^ (https://www.medscinet.net/snr/), we identified 9111 dialysis patients who started dialysis between January 1, 2005 and December 31, 2015. After excluding patients whose age was <18 years at 1 year before initiation of dialysis (*n* = 70), we included 9041 patients in the present study. SRR provided demographic data (age, gender, cause of end‐stage kidney disease [ESKD], date of dialysis initiation, and kidney transplantation date), which was linked to national administrative databases containing complete information on each individual's health care use, dispensed drugs, diagnosis, and clinical outcomes.^(^ ^18^ ^)^ All linkages were performed centrally and anonymized by the Swedish National Board of Welfare. The Ethics Committee of the Karolinska Institutet, Stockholm, Sweden, approved study protocols.

### Major fractures

After linkage with the Swedish National Patient Register where a primary diagnosis is listed in 99% of all hospital discharges in Sweden, we identified all cases who had a primary diagnosis of a major fracture affecting hip, spine, humerus, or forearm, based on the International Classification of Diseases and Related Health Problems, 10th Revision (ICD‐10) codes (Supplemental Table 1) from 1 year before until 1 year after dialysis initiation. We included only cases where fracture was stated as the primary diagnosis because we aimed at capturing the incidence of fractures occurring during normal conditions rather than fractures related with uncommon situations such as a traffic accident.

Those with fracture occurring prior to 1 year before dialysis initiation were classified as having history of previous major fractures.

### Study covariates

Study covariates at 1 year before initiation included age, gender, comorbidities, and concomitant medications. Comorbid conditions included diabetes mellitus, cancer (within 5 years prior to 1 year before initiation), previous history of major fractures, dementia, ischemic heart disease, congestive heart failure, peripheral vascular disease, cerebrovascular disease, hyperparathyroidism, and psychoactive substance abuse, assessed using the ICD‐ 10 (Supplemental Table 1). Diagnostic codes are available since the implementation of ICD‐10 in Sweden in 1997. Medications included renin–angiotensin–aldosterone system (RAAS) inhibitors, vitamin D and its analogues, phosphate binders, estrogen, statins, anti‐anxiolytics drugs, steroids, and anti‐depressive drugs (Supplemental Table 2). Information on drug dispensations was obtained from the Dispensed Drug Registry, a nationwide register with complete information on all prescribed drugs dispensed at Swedish pharmacies.^(^ ^19^ ^)^ Drugs were assumed to be used if there was a pharmacy dispensation within 6 months prior to 1 year before dialysis initiation.

### Statistical analyses

Data are expressed as median (25th to 75th percentile) or percentage or subdistribution hazard ratio (sHR) or incidence rate, as appropriate. Statistical significance was set at the level of *p* < .05. Statistical analyses were performed using Stata 16.0 (StataCorp LLC, College Station, TX, USA) and SAS version 9.4 (SAS Institute, Inc., Cary, NC, USA).

#### Analyses of incident rate of fractures

We followed patients from 1 year before dialysis initiation until the occurrence of the first fracture, kidney transplantation, death, or completion of follow‐up 1 year after dialysis initiation, whichever came first. We calculated the crude incidence rate of fractures every month from 1 year before dialysis initiation. We used a multivariate flexible parametric survival model^(^ ^20^ ^)^ accounting for competing risk of death and kidney transplantation to assess the temporal development of incident fracture rates as a time‐dependent variable. We adjusted for sex, age, history of major fractures, comorbidities (diabetes mellitus, cancer, dementia, ischemic heart disease, congestive heart failure, peripheral vascular disease, cerebrovascular disease, and hyperparathyroidism), medications (psychoactive substance abuse, RAAS inhibitors, vitamin D and its analogues, phosphate binders, estrogen, statin, anti‐anxiolytics, steroids, anti‐depressives), and calendar year of dialysis initiation,

#### Prediction of first major fractures following dialysis initiation

Fine and Gray competing risk regression analysis was used for prediction of major fractures. Thus, we estimated the sHR, taking death and kidney transplantation into account as a competing risk to prevent overestimation of fracture risk.^(^ ^21^, ^22^ ^)^ This analysis was done separately for fracture incidence before dialysis initiation and fracture incidence after dialysis initiation, adjusting for sex, age, history of major fractures, comorbidities (diabetes mellitus, cancer, dementia, ischemic heart disease, congestive heart failure, peripheral vascular disease, cerebrovascular disease, and hyperparathyroidism), medications (psychoactive substance abuse, RAAS inhibitors, vitamin D and its analogues, phosphate binders, estrogen, statins, anti‐anxiolytics, steroids, anti‐depressives), and calendar year of dialysis initiation.

## Results

### Baseline characteristics

Table 1 shows demographics and clinical characteristics at 1 year before dialysis initiation. The median age was 67 years, 67% were men, and 6.3% had history of previous major fractures. When patients were divided into those with and without major fractures occurring during 1 year before and up to 1 year after dialysis initiation, those with major fractures had higher age, a higher proportion were women, and a history of major fractures, cancer, congestive heart failure, cerebrovascular disease, and psychoactive substance abuse were more common. Anti‐depressive drugs were more often prescribed in those with major fractures.

**Table 1:** Baseline Characteristics of 9041 Incident Dialysis Patients at 1 Year Before Dialysis Initiation

Characteristic	Total	Patients with major fractures	Patients without major fractures	*p*
Sample size, *n* (%)	9041 (100)	361 (4)	8680 (96)	
Sex, female, *n* (%)	2990 (33.1)	172 (47.6)	2818 (32.5)	<.001
Age (years), median (IQR)	67 (55–75)	70 (64–79)	66 (55–75)	<.001
Age category, *n* (%)				<.001
<45 years	1118 (12.4)	12 (3.3)	1106 (12.7)	
45–64 years	2875 (31.8)	85 (23.5)	2790 (32.1)	
≥65 years	5048 (55.8)	264 (73.1)	4784 (55.1)	
Previous history major fractures, *n* (%)	571 (6.3)	71 (19.7)	500 (5.8)	<.001
Primary cause of ESKD, *n* (%)				<.05
CGN	1180 (13.1)	28 (7.8)	1152 (13.3)	
DN	2420 (26.8)	112 (31.0)	2308 (26.6)	
HT/RVD	1399 (15.5)	56 (15.5)	1343 (15.5)	
Other	4042 (44.7)	165 (45.7)	3877 (44.7)	
Comorbidity, *n* (%)				
Diabetes	3567 (39.5)	160 (44.3)	3407 (39.3)	.053
Cancer	1968 (22.7)	116 (32.5)	1852 (22.2)	<.001
Dementia	27 (0.3)	2 (0.6)	25 (0.3)	.36
Ischemic heart disease	1627 (18.0)	74 (20.5)	1553 (17.9)	.21
Congestive heart failure	1512 (16.7)	76 (21.1)	1436 (16.5)	<.05
Peripheral vascular disease	1022 (11.3)	48 (13.3)	974 (11.2)	.22
Cerebrovascular disease	1189 (13.2)	63 (17.5)	1126 (13.0)	<.05
Hyperparathyroidism	231 (2.6)	9 (2.5)	222 (2.6)	.94
Psychoactive substance abuse	433 (4.8)	26 (7.2)	407 (4.7)	<.05
Medications, *n* (%)				
RAAS inhibitor	3275 (36.2)	133 (36.8)	3142 (36.2)	.80
Vitamin D and its analogues	2472 (27.3)	97 (26.9)	2375 (27.4)	.84
Phosphate binder	763 (8.4)	35 (9.7)	728 (8.4)	.38
Estrogen	130 (1.4)	8 (2.2)	122 (1.4)	.20
Statin	2321 (25.7)	103 (28.5)	2218 (25.6)	.20
Anti‐anxiolytics	556 (6.1)	30 (8.3)	526 (6.1)	.081
Steroid	650 (7.2)	31 (8.6)	619 (7.1)	.29
Anti‐depressive	604 (6.7)	42 (11.6)	562 (6.5)	<.001
Calendar year dialysis initiation, *n* (%)				.11
2014–2015	1737 (19.2)	70 (19.4)	1667 (19.2)	
2011–2013	2459 (27.2)	94 (26.0)	2365 (27.2)	
2008–2010	2503 (27.7)	118 (32.7)	2385 (27.5)	
2005–2007	2342 (25.9)	79 (21.9)	2263 (26.1)	

Continuous variables are presented as median (25th to 75th percentile). Categorical variables are presented as number (*n*)/percentage (%).

CGN = chronic glomerulonephritis; DN = diabetic nephropathy; ESKD = end‐stage kidney disease; HT = hypertension; IQR = interquartile range; RAAS = renin–angiotensin–aldosterone system; RVD = renal vascular disease.

### Incidence of fractures before and after dialysis initiation

**Fig 1:**
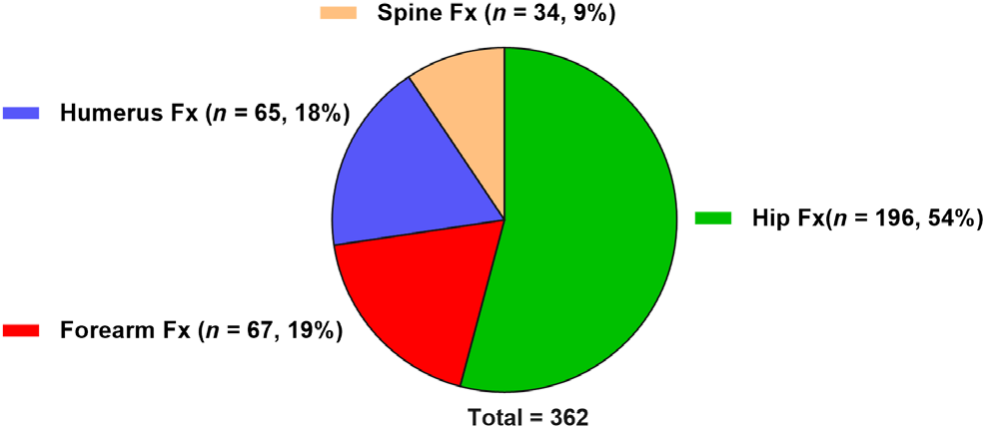
Location of the first Fx occurring from 1 year before dialysis initiation to 1 year after dialysis initiation. Fx = fracture.

**Fig 2:**
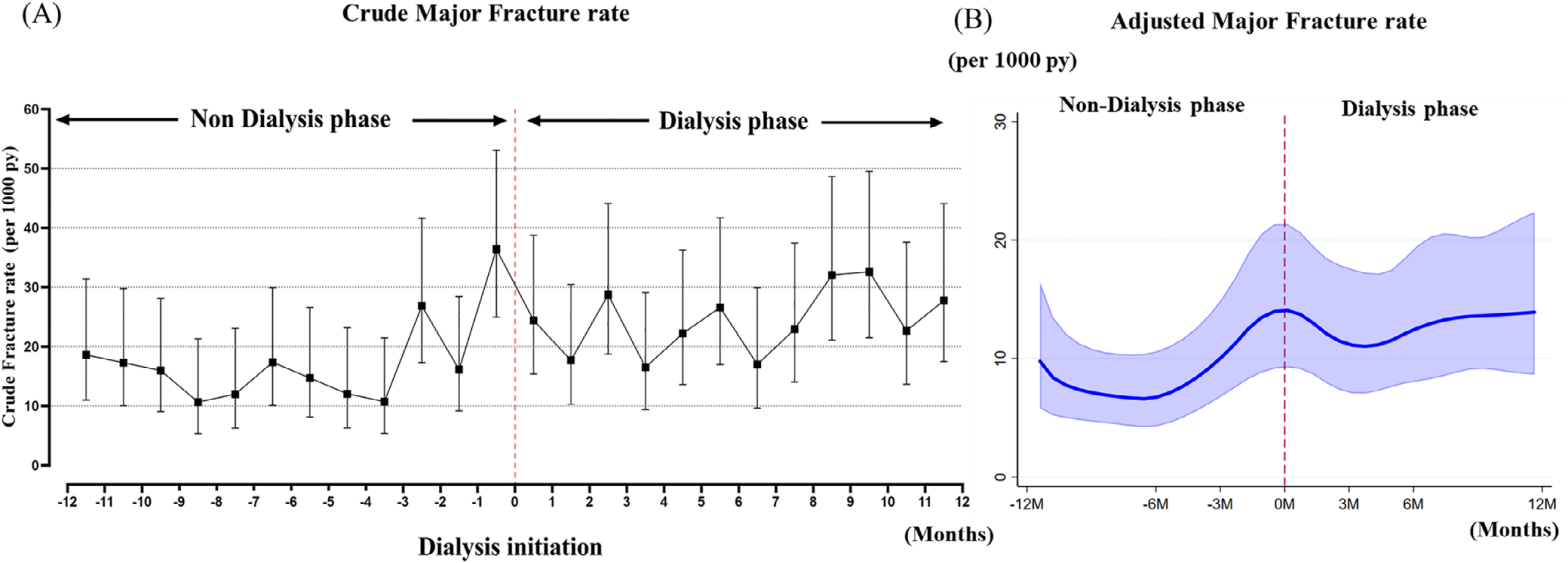
Crude (*A*) and adjusted (*B*) incidence rate of first major fracture occurring from 12 months before until 12 months after dialysis initiation. The adjusted incidence rate of fracture was calculated using flexible parametric survival models in hypothetical 67‐year‐old patient (median age) after adjusting for gender, age, history of major fractures, diabetes mellitus, cancer, dementia, ischemic heart disease, congestive heart failure, peripheral vascular disease, cerebrovascular disease, hyperparathyroidism, psychoactive substance abuse, RAAS inhibitor, vitamin D and its analogues, phosphate binders, estrogen, statins, anti‐anxiolytics, steroids, anti‐depressives, and calendar year of dialysis initiation. py = patient‐years.

**Fig 3:**
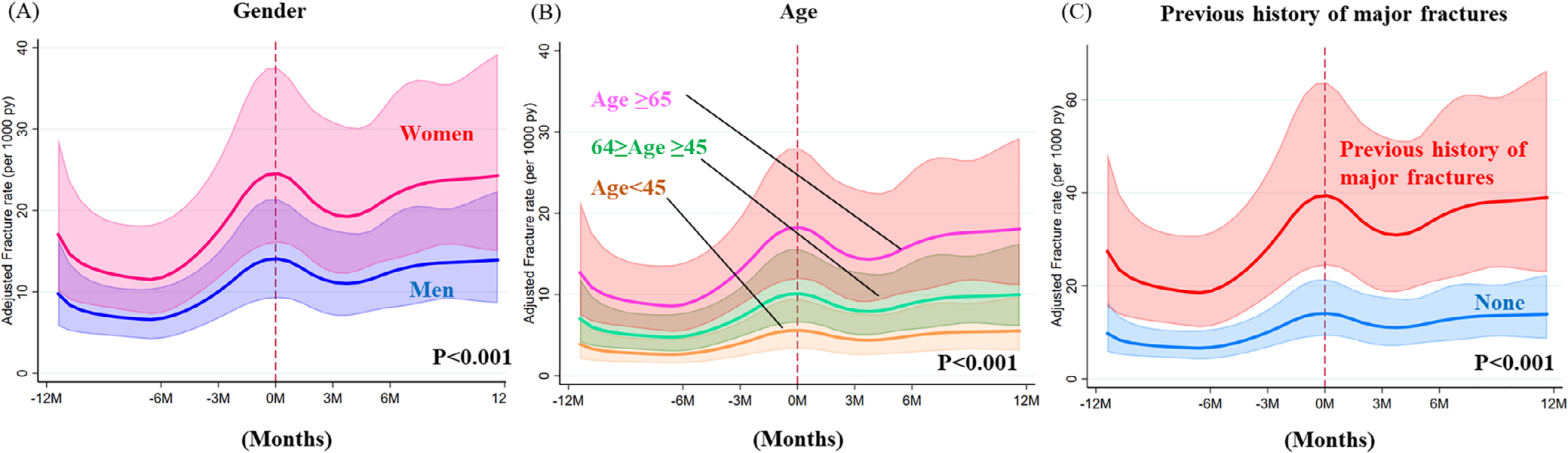
Impact of gender (*A*), age (*B*), and history of previous major fractures (*C*) on adjusted incidence rate of major fractures expressed as fractures per 1000 py occurring from 12 months before until 12 months after dialysis initiation. The adjusted incidence rate of fractures was calculated using flexible parametric survival models in a hypothetical 67‐year‐old patient (median age) after adjusting for diabetes mellitus, cancer, dementia, ischemic heart disease, congestive heart failure, peripheral vascular disease, cerebrovascular disease, hyperparathyroidism, psychoactive substance abuse, RAAS inhibitors, vitamin D and its analogues, phosphate binders, estrogen, statins, anti‐anxiolytics, steroids, anti‐depressives, and calendar year of dialysis initiation. py = patient‐years.

During follow‐up, there were 361 fractures in total (4% of all 9041 patients), of which 196 were hip fractures (Fig. 1). During follow‐up, 404 patients underwent kidney transplantation and 587 patients died. The crude incidence rate of major fractures before (*n* = 157) initiation was 17 per 1000 patient‐years and after (*n* = 204) initiation it was 24 per 1000 patient‐years. The crude incidence rate of major fractures increased during the months before initiation of dialysis, peaked at a rate of 36 per 1000 patient‐years at 1 month before initiation, and decreased 2 months after initiation, to finally stabilize at a higher rate than during the pre‐dialysis phase (Fig. 2*A*). The multivariate‐adjusted fracture incidence rates derived from the multivariate flexible parametric survival model began rising earlier before initiation of dialysis, peaked just before initiation of dialysis, and eventually stabilized. Overall, the fracture rate in the dialysis phase was higher than the initial rate during the non‐dialysis phase (Fig. 2*B*). Figure 3*A–C* shows that female sex, higher age, and having a history of major fractures were associated with higher risk of fracture events.

### Type of fracture

**Fig 4:**
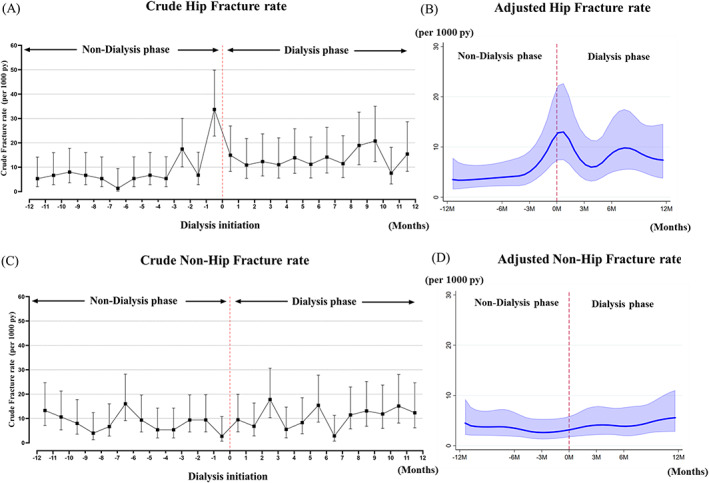
Crude and adjusted incidence rate of hip fractures (*A*,*B*) and non‐hip fractures (*C*,*D*) occurring from 12 months before until 12 months after dialysis initiation. Adjusted incidence rate of fracture was made using flexible parametric survival models in hypothetical 67‐year‐old patient (median age) after adjusting for gender, age, history of major fractures, diabetes mellitus, cancer, dementia, ischemic heart disease, congestive heart failure, peripheral vascular disease, cerebrovascular disease, hyperparathyroidism, psychoactive substance abuse, RAAS inhibitor, vitamin D and its analogues, phosphate binders, estrogen, statins, anti ‐anxiolytics, steroids, anti‐depressives, and calendar year of dialysis initiation. py = patient‐years.

Among 361 major fractures events, 196 (54%) were fractures of the hip and the remaining 165 (46%) were grouped as “non‐hip fracture” (forearm, humerus, and spine fracture). For hip fractures, 82 fractures (9/1000 patient‐years) occurred in the non‐dialysis phase and 114 fractures (13.5/1000 patient‐years) occurred during the dialysis phase. The crude hip fracture incidence rate rapidly increased approximately 3 months before initiation of dialysis, peaked at 34 cases per 1000 patient‐years the last month before dialysis initiation, then decreased after initiation, and stabilized with a higher rate than at 1 year before dialysis initiation (Fig. 4*A*). A similar trajectory was observed for adjusted hip fracture incidence rate (Fig. 4*B*). For non‐hip fractures, 75 fractures (8.3/1000 patient‐years) occurred in the non‐dialysis phase and 90 fractures (10.8/1000 patient‐years) during the dialysis phase. The trajectories of both crude and adjusted non‐hip fracture rates appeared to be rather stable before and after dialysis initiation although both showed a gradual increase over time (Fig. 4*C*,*D*).

### Determinants of fracture incidence before and after dialysis initiation

Female sex, higher age, previous history of major fractures, and cancer were found to be associated with increased fracture risk. Medications and other comorbidity conditions including diabetes mellitus were not associated with an increased risk of major fractures (Table 2).

**Table 2:** Risk Factors Associated With Fractures Occurring Before and After Dialysis Initiation

	Fracture before initiation		Fracture after initiation	
Characteristic	sHR (95% CI)	*p*	sHR (95% CI)	*p*
Sex (women versus men)	1.91 (1.38–2.64)	<.001	1.60 (1.19–2.16)	<.01
Age category (ref: <45 years)				
45–64 years	4.10 (1.26–13.39)	<.05	1.93 (0.91–4.11)	.087
≥65 years	6.59 (2.08–20.88)	<.01	3.20 (1.55–6.60)	<.01
Previous history major fractures	3.59 (2.43–5.31)	<.001	2.31 (1.55–3.44)	<.001
Diabetes mellitus	1.09 (0.77–1.52)	.633	1.09 (0.80–1.47)	.598
Cancer	1.43 (1.00–2.03)	.050	1.49 (1.11–2.01)	<.01
Dementia	2.23 (0.28–18.01)	.450	1.39 (0.18–10.8)	.756
Ischemic heart disease	1.11 (0.73–1.70)	.626	0.90 (0.63–1.30)	.583
Congestive heart failure	0.93 (0.60–1.43)	.735	1.14 (0.81–1.60)	.463
Peripheral vascular disease	0.92 (0.57–1.47)	.715	0.94 (0.61–1.44)	.763
Cerebrovascular disease	1.01 (0.65–1.58)	.952	1.21 (0.84–1.76)	.302
Hyperparathyroidism	1.00 (0.41–2.42)	.998	0.65 (0.25–1.73)	.389
Psychoactive substance abuse	1.65 (0.90–3.01)	.103	1.57 (0.90–2.73)	.113
RAAS inhibitor	0.87 (0.61–1.24)	.845	1.03 (0.77–1.37)	.845
Vitamin D and its analogues	0.98 (0.67–1.42)	.911	0.78 (0.56–1.08)	.138
Phosphate binder	1.13 (0.65–1.96)	.657	1.13 (0.70–1.83)	.618
Estrogen	0.25 (0.03–1.75)	.162	1.62 (0.73–3.56)	.233
Statin	1.06 (0.72–1.56)	.763	1.08 (0.78–1.50)	.627
Anti‐anxiolytics	1.32 (0.80–2.17)	.281	0.80 (0.45–1.44)	.081
Steroid	1.03 (0.57–1.85)	.919	1.09 (0.67–1.76)	.722
Anti‐depressive	1.53 (0.95–2.47)	.084	1.46 (0.93–2.30)	.100
Patient recruitment year (ref: 2014–2015)				
2011–2013	0.96 (0.58–1.59)	.574	1.13 (0.75–1.70)	.574
2008–2010	1.58 (0.99–2.83)	.056	1.21 (0.80–1.85)	.369
2005–2007	0.99 (0.57–1.71)	.965	1.07 (0.68–1.69)	.759

CI = confidence interval; RAAS = renin–angiotensin–aldosterone system; sHR = subdistribution hazard ratio.

#### Factors associated with fractures before initiation of dialysis

Female sex (sHR 1.9 [versus male], *p* < .001), higher age (age ≥ 65 years: sHR 6.59 [versus age <45 years], *p* < .01), age between 45 and 64 years (sHR 4.10 [versus age <45 years], *p* < .05), and previous history of major fractures (sHR 3.59 [versus no history of major fractures], *p* < .001) were associated with fractures before dialysis initiation.

#### Factors associated with fractures after initiation

Female sex (sHR 1.6 [versus male], *p* < .01), higher age (age ≥ 65 years: sHR 3.20 [versus age < 45 years], *p* < .01), previous history of major fractures (sHR 2.31 [versus no history of major fractures], *p* < .001), and presence of cancer (sHR 1.49 [versus no presence of cancer], *p* < .01) were associated with fractures after dialysis initiation.

## Discussion

In this nationwide, population‐based cohort study of incident dialysis patients starting dialysis in 2005 through 2015 in Sweden, we investigated the trajectory of fracture rate for different types of osteoporotic fractures occurring from 1 year before until 1 year after dialysis initiation. Our study shows that major fractures affect 4% of incident dialysis patients, and that the fracture rate begins to increase already at 6 months before dialysis initiation. After dialysis initiation, the fracture incidence becomes stabilized, but at a higher level than 1 year before dialysis. The trajectory, however, differed for the various types of major fractures. Although hip fracture incidence rose already 3 months before initiation, peaked close to the initiation of dialysis, and then stabilized on a higher level than before dialysis, the trajectory of non‐hip fractures was similar before and after dialysis initiation although with a gradually increasing incidence over time. Furthermore, we identified some factors that associated with significantly increased fracture risk including being a woman, high age, history of previous major fractures, and cancer.

To the best of our knowledge, this is the first study that describes how fracture incidence changes over the dialysis transition period from 1 year prior to dialysis initiation until 1 year after dialysis initiation. The transition period from non‐dialysis phase to dialysis phase is critical because dialysis initiation usually occurs under the worst health conditions. In this period, worsening of uremic symptoms including fatigue, anorexia, nausea, fluid overload with dyspnea, anemia, and other laboratory changes due to uremia including electrolyte and mineral disturbances such as hyperkalemia and acidosis, are factors that necessitate start of dialysis.^(^ ^23^, ^24^, ^25^ ^)^

These and numerous other factors contribute to high cardiovascular event rates and markedly increased mortality risk during the dialysis transition period^(^ ^26^, ^27^, ^28^ ^)^ and may have also contributed to the high fracture incidence rate observed in the current study. The higher incidence of fracture in dialysis patients compared to non‐dialysis–dependent CKD patients^(^ ^3^ ^)^ may thus reflect changes starting already during the dialysis transition period. These changes include deterioration of physical and cognitive functions, worsening of complications related to CKD‐MBD such as vascular calcification and increased bone fragility due to osteoporosis/renal osteodystrophy that together with increased hemodynamic instability and propensity for falls conceivably lead to an increased fracture risk.

Interestingly, our finding that the fracture rate began to rise already before dialysis initiation concurs with results from a previous study in the US focusing on stroke events during the transition period: among incident dialysis patients (age ≥ 67 years), the incidence rate of stroke started to increase a few months before dialysis initiation both in outpatient and inpatient initiators, regardless of dialysis modality.^(^ ^29^ ^)^ The authors concluded that the process of initiating dialysis may lead to strokes, whereas considering the possibility that stroke itself may lead to ESKD via complications associated with stroke such as sepsis.

Although the etiology of stroke and fractures in patients with advanced CKD is not similar, a rapid worsening of physical functions assessed by degree of seven daily living activities was reported to begin already 3 months before initiation of dialysis.^(^ ^8^ ^)^ Besides, the incidence of serious falls gradually increased from 1 year before starting dialysis and peaked at dialysis initiation in a study using data from the US Renal Data System (USRDS).^(^ ^9^ ^)^ These two factors, deterioration of physical functions and serious falls, are well known contributors to an increased fracture incidence both in the general population and in CKD patients^(^ ^30^, ^31^, ^32^ ^)^ and may have hastened fracture incidence in the present study. However, because these factors should influence the different types of fractures similarly, this does not explain why trajectories for hip fractures and non‐hip fractures differed in our study. However, although hip fractures are often accompanied by a general deterioration of the patient's condition including worsening of anemia, sometimes requiring blood transfusion, further loss of kidney function, acidosis, and mineral and electrolyte disorders (especially hyperkalemia),^(^ ^33^, ^34^ ^)^ these phenomena may be less pronounced following non‐hip fractures. It is possible that these and other factors may affect the fracture types differently. For instance, body composition changes including reduced adiposity prior to dialysis initiation,^(^ ^35^ ^)^ possibly conferring an increase hip fracture risk, may boost hip fracture incidence, not non‐hip fracture incidence. Another possible explanation for the different trajectory between hip fracture and non‐hip fracture is that hip fracture may be a rationale to initiate dialysis therapy in some patients.

Our study is an observational study by design and as such does not allow conclusion about a causal relationship between fracture incidence and dialysis initiation. However, our findings of a high hip fracture incidence rate from 3 months before and after dialysis initiation may alert clinicians that the dialysis transition period is a vulnerable period during which vigilant medical surveillance with implementation of preventive measures is warranted.

We observed that high age, female sex, history of previous fracture, and cancer were important predictors of the incidence of major fractures. In addition, psychoactive substance abuse and use of anti‐depressive drugs also appeared to have a negative impact on fracture events, though these associations did not reach statistical significance in our analysis. Of note, diabetes mellitus was not associated with fracture events, neither before nor after initiation of dialysis, which is in line with results of a previous study using the second phase of the Dialysis Outcomes and Practice Patterns Study (DOPPS).^(^ ^36^ ^)^ In contrast, diabetes mellitus is a known risk factor for fractures in the general population and among kidney transplant recipients.^(^ ^37^, ^38^, ^39^, ^40^ ^)^

In our analysis, cancer was an independent predictor for fractures occurring after initiation of dialysis. The aging population and major advances in the treatment for cancer has led to an increasing number of people who live longer with their cancer. Although decision making for dialysis initiation in CKD patients with cancer can be complicated, cancer is in general not a contraindication to dialysis.^(^ ^41^ ^)^ The high percentage of patients, 22% in our study, with history of cancer diagnosis within 5 years prior to 1 year before dialysis initiation probably reflects both the high median age of dialysis patients and the increased risk for cancer that accompanies a decline of renal function.^(^ ^42^, ^43^ ^)^ The high burden of cancer among patients with ESKD and our observation that fracture risk is increased in dialysis patients with cancer diagnosis underline that improved surveillance to prevent fractures is warranted in these patients.

The strengths of this study include the use of a national register of dialysis patients in a country with access to universal healthcare system and linkages to other national databases provided exact information on type and date of fractures, dispensed drugs, causes of deaths, and kidney transplantation. In addition, we had information about previous fractures and time of occurrence of first fracture from 1 year before dialysis to 1 year after dialysis initiation, and no patients were lost to follow‐up. For detecting fracture incidence, our database (SRR) was linked to the Swedish National Patient Register, comprising data on all health care episodes in inpatient and outpatient specialist care in Sweden, where a primary diagnosis is listed in 99% of all hospital discharges in Sweden. Compared to the Swedish Hip Fracture Registry, the proportion of all hip fracture cases identified through the Swedish National Patient Register is 95.3%. The positive predictive value of hip fracture diagnosis in the Swedish National Patient Register is 95% to 98.4%.^(^ ^18^ ^)^ Thus, the Swedish National Patient Register is a useful tool for hip fracture research in Sweden because of high validity and completeness of data. We could capture any fracture, regardless of hospitalization or not, and thus the generalizability is high although confined to the situation in Sweden, with a mainly white population. To prevent overestimation of the risk of fractures, we performed a competing risk analysis taking the competing risks of death and kidney transplantation into account.^(^ ^21^, ^22^ ^)^

Some limitations should be considered when interpreting the results. First, as in any observational study, we cannot conclude on causality. Furthermore, many important confounders were not accounted for such as biochemical markers, bone histological findings, bone mineral density, health status such as frailty index, alcohol consumption, and smoking, which most likely may have affected the risk of fracture. Especially, the absence of biomarkers related with CKD‐MBD (ie, calcium, phosphate, PTH, and 25‐OH‐vitamin D) that may influence bone health and thus fracture risk is a weakness. To some extent, the use of a national database and extensive linkages to other healthcare sources may indirectly compensate for this. For instance, psychoactive substance abuse detected by ICD code may indirectly at least to some extent reflect the impact of heavy alcohol consumption and smoking. Second, the incidence of spine fractures using data from administrative databases, and not from radiographic and medical charts, may be underestimated because some fractures of the spine are nontraumatic and often go undiagnosed in clinical practice. Third, because our aim was to investigate how fracture incidence was changed before and after dialysis initiation, we were unable to include patients who developed fatal fractures in the non‐dialysis phase and therefore could not reach dialysis initiation because our inclusion criteria were incident dialysis patients. This potential bias may lead to underestimated fracture incidence in the non‐dialysis phase especially for hip fractures because of the higher mortality following hip fractures than after non‐hip fractures.^(^ ^15^ ^)^ Our results suggest that clinicians need to be especially aware of risk of hip fractures during the dialysis transition period.

In conclusion, in ESKD patients initiating dialysis, the incidence rate of major fractures started to increase from 6 months before initiation of dialysis, and there was a further increase following dialysis initiation. There was a marked difference in trajectories for hip fractures and non‐hip fractures: the incidence of hip fractures started to increase markedly 3 months before dialysis initiation, whereas no such high‐risk period prior to dialysis was observed for non‐hip fractures. These findings suggest that heightened surveillance is warranted in patients with CKD stage G5, especially in older women with a history of previous major fracture, to prevent future hip fractures. However, the external validity to other countries than Sweden should also be investigated.

## Disclosures

BL is employed by Baxter Healthcare Corporation. PS reports personal fees from REATA, Baxter, Fresenius Medical Care, Astellas, Astra Zeneca, and Pfizer, outside the submitted work. ME reports personal fees for lectures (Vifor Pharma, Astellas, Fresenius Medical Care), advisory board (Astra Zeneca, Astellas, Vifor Pharma), and travel grant (Baxter), outside the submitted work. None of the other authors declare any conflict of interest.

### Peer Review

The peer review history for this article is available at https://publons.com/publon/10.1002/jbmr.4141.

## Supporting information


**Supplemental Table 1** Definition of comorbidities and outcomes by ICD‐10 codes.
**Supplemental Table 2** Definition of medications by Anatomical Therapeutic Chemical (ATC) Classification codes.
