# Supplementary material for: Incidence of Fractures Before and After Dialysis Initiation
Source: J Bone Miner Res. 2020 Sep 16;35(12):2372–80. doi: 10.1002/jbmr.4141 (PMC7757394; doi:10.1002/jbmr.4141)
Supplement: Supplementary file 1 — Supplemental Table 1 Definition of comorbidities and outcomes by ICD‐10 codes. Supplemental Table 2 Definition of medications by Anatomical Therapeutic Chemical (ATC) Classification codes. [file JBMR-35-2372-s001.docx]

**Supplemental Table 1.** Definition of comorbidities and outcomes by ICD-10 codes.

| **Variable** | **ICD-10 codes** |
| --- | --- |
| ***Major Fractures*** |  |
| Hip fracture | S720, S721, S722 |
| Non-hip fracture (forearm, spine, humerus) | S220, S221, S320, S327, S328, S422, S525, S526 |
| ***Comorbidities*** |  |
| Diabetes mellitus | E10-E14 |
| Cancer | C |
| Dementia | F00-F03, F051, G30, G311 |
| Ischemic heart disease | I21- I25 |
| Congestive heart failure | I099, I110, I130, I132, I255, I420, I425-429, I43, I50, P290 |
| Peripheral vascular disease | I70, I71, I731, I738, I739, I771, I790, I792, K551, K558, K559 |
| Cerebrovascular disease | G45-46, H341, I6 |
| Hyperparathyroidism | E21 |
| Psychoactive substance abuse | F10-F19 |

**Supplemental Table 2.** Definition of medications by Anatomical Therapeutic Chemical (ATC) Classification codes.

| **Medication** | **ATC codes** |
| --- | --- |
| Renin-angiotensin-aldosterone system inhibitors | C09A-D, C03DA |
| Vitamin D and its analogues | A11CC |
| Phosphate binders | V03AE02, V03AE03 |
| Estrogen supplementation | G03C |
| Statins | C10AA |
| Anti-anxiolytics | N05B |
| Steroid | H02A, H02B |
| Anti-depressive | N06A |
